# Supplementary material for: Patterns of Intron Gain and Loss in Fungi
Source: PLoS Biol. 2004 Nov 30;2(12):e422. doi: 10.1371/journal.pbio.0020422 (PMC532390; doi:10.1371/journal.pbio.0020422)
Supplement: Table S1 — Also available at http://genes.mit.edu/NielsenEtAl/. (4.3 MB ZIP). [file pbio.0020422.st001.zip › NielsenEtAl/html/98.html]

AN6976.1.NCU01859.1.MG09006.1.FG11619.1


```
 CLUSTAL W (1.82) Multiple Sequence Alignments - Introns Inserted


Sequence 1: NCU01859.1	210 aa
Sequence 2: MG09006.1	189 aa
Sequence 3: FG11619.1	187 aa
Sequence 4: AN6976.1	189 aa
Alignment Length: 215 aa
Number Identitical Residues: 97 aa
Alignment Score (without introns) 4713


MG09006.1 	----MSIDSTARYV-PSKTLPSEYP0LIDNDP2HFKRVVSYARPSDYAYGATAAAAGPGL
NCU01859.1	----MSSTSSPTYT-ISKTLNTNYP0LIDNDP2HFRRVIGYARPSDYVHGTVAGAAGPGL
FG11619.1 	MAETATKQATPSFVGTSKVVQTDYP0LIDNDP2HFKRVVGYARTSDYLAGTAAAAFAPAA
AN6976.1  	----MAGVNNSTVV-PPKRALTDYP0LIDSDP2HVRRVFGYARPSDYAVGAGAAAASPLA
          	     :   ..  .  .*   ::** ***.** *.:**..***.***  *: *.* .*  

MG09006.1 	LTLMEKVSPSLVGRGGFAQAMRLTGAVGLTGGFLLFYQRSCL1RFFGMSENSREVDMDMR
NCU01859.1	LYLMEKMAPSGVGKGGFPKAMRLATAVGFFGGFLYFYQRSIL1RFYGMSENAREVQMDMR
FG11619.1 	LYALEKFAPSHVGKGGFAKAMRLAGGIGVLGGFLYFYQRSAL1RFYGATENSREIEMDMR
AN6976.1  	FWLMERVSPSHVGRGGFSPVMRLATAIGLIGGVHVLYQRSCN1RFHGFTENSREVEMDMR
          	:  :*:.:** **:***. .***: .:*. **.  :****   **.* :**:**::****

MG09006.1 	EMVDKVKKGEPLYGVSQLTPNMQGVAARQSRYSALFHEVVPWFNFVNHNQ~HGVDTAKYY
NCU01859.1	EMVDKVKAGQPLYGVSTLPVDVQGMAARQSRYSALFFAVLPWFNFVNHNQ~HGVDTAKYY
FG11619.1 	EMVDKVKKGEPLYGASRLSPHLQGVAARQSRYSALFLSTIPWFNFVNHNQ~HGVDTAKYY
AN6976.1  	EMVDKVKKGEPLYGASQVSPHLQGVAARNSRYANLFIQVIPWFNLVNHEH0HGVDTAKYY
          	******* *:****.* :. .:**:***:***: **  .:****:***:: *********

MG09006.1 	RQAERELAAEGKTLKESS~---------------------
NCU01859.1	QQAERELEAERLGKGSSS2TERKLTTIPYPDLYVILQEVR
FG11619.1 	QQAERELEAER-------~---------------------
AN6976.1  	QQAERELEAERLSKLSSS~---------------------
          	:****** **     .::
```
